# Microwaves increase the effectiveness of systemic antibiotic treatment in acute bone infection: experimental study in a rat model

**DOI:** 10.1186/s13018-019-1342-3

**Published:** 2019-09-06

**Authors:** Xiao-yang Qi, Xu-sheng Qiu, Jiang-yun Jiang, Yi-xin Chen, Li-ming Tang, Hong-fei Shi

**Affiliations:** 1 Department of Orthopaedics, Nanjing Drum Tower Hospital, Clinical College of Nanjing Medical University, 321 Zhongshan Road, Nanjing, China; 2 Department of Orthopaedics, Nanjing Drum Tower Hospital, The Affiliated Hospital of Nanjing University Medical School, 321 Zhongshan Road, Nanjing, China; 3 Department of Surgery, The Affiliated Changzhou No. 2 People’s Hospital of Nanjing Medical University, 68 Gehu Road, Changzhou, China

**Keywords:** Microwave, Osteomyelitis, *Staphylococcus aureus*, Cefuroxime

## Abstract

**Background:**

Osteomyelitis is a challenge for orthopedic surgeons due to its protracted treatment process. Microwaves (MWs) can increase blood perfusion due to their thermal effect. Furthermore, MWs demonstrated significant bactericidal effects in vitro. In the present study, we assumed that the application of a 2450-MHz-frequency MW together with systemic antibiotic treatment would provide synergy for the treatment of acute osteomyelitis.

**Methods:**

The medullary cavity of the right tibia was inoculated with 10^7^ CFU of methicillin-sensitive *Staphylococcus aureus* (MSSA-ATCC 29213) in 40 rats, and the rats were randomly divided into four groups according to treatment: group I, saline (control); group II, saline + MW therapy; group III, systemic cefuroxime; and group IV, systemic cefuroxime + MW therapy. MWs were applied for 20 min per day to the infected limbs, and all rats were sacrificed on the 7th day. The severity of tibial osteomyelitis was assessed by quantitative culture analysis.

**Results:**

Bacterial counts in groups III and IV were significantly reduced compared with those in the control (*p* = 0.001 and < 0.001, respectively). Furthermore, significant differences were detected between groups III and IV (*p* = 0.033). However, the difference between groups I and II was nonsignificant (*p* = 0.287).

**Conclusion:**

Our experimental model suggests that MW therapy provides a significant synergy for systemic antibiotic treatment. However, further clinical trials are required to safely use this treatment modality in patients.

## Introduction

Osteomyelitis is an inflammatory disorder of the bone caused by infection [1]. It is still challenging for orthopaedic surgeons because of its protracted treatment process. The reported infection control rate ranges from 67 to 95% [2], which means that 5% to 33% of patients suffer from recurrence of infection. In recent years, some new adjuvant treatments have been attempted to treat and cure osteomyelitis, for example, hyperbaric oxygen therapy, pulsed electromagnetic fields, ultrasound, laser, and extracorporeal shockwave, which have been demonstrated with successful results in the treatment of osteomyelitis [3–6]. Despite advances in surgical treatment, antibiotic therapy, diagnostic methods, and differentiated approaches to each type of osteomyelitis, the treatment outcomes are still unsatisfactory [7].

As we know, the local blood supply to the bone is impaired in patients with osteomyelitis [8–10]; therefore, strategies to improve blood supply and tissue perfusion will be beneficial to improve microbial clearance in infected areas and minimize recurrence in predisposed regions. The approaches to achieve this goal have typically been surgical, with transfer of tissue flaps and adequate debridement [10]. Microwaves (MWs) are non-ionizing electromagnetic waves with frequencies ranging from 300 to 300 GHz. The tissue can absorb the MW energy as vibrational energy. Due to the nature of the tissue, this vibrational energy is converted into perceivable warmth in the tissue. Hyperthermia induced by MW diathermy can increase the temperature of deep tissues, which contributes to a regional increase in blood flow. Effective clinical responses occur when the tissue temperature reaches 41 to 45 °C, and the blood perfusion increases by approximately 15 times [11]. Therefore, MWs may theoretically beneficial to the treatment of osteomyelitis. Furthermore, the MWs delivered from Prostatron version 2.0 (a computer-driven system, working frequency: 1296 MHz) with temperature no more than 44 °C showed a bactericidal effect in vitro on laboratory-cultured *Escherichia coli* and *Enterobacter cloacae* [12]. Considering the increased blood perfusion induced by MW diathermy and the possible bactericidal effect of MWs, a hypothesis was proposed that MWs may be an easy and effective adjuvant therapy for the prevention or treatment of osteomyelitis. Therefore, the present study aimed to investigate whether MWs may play a role in the treatment of acute bone infection in a rat model. The influence of MWs and a combination of MWs and systemic antibiotic treatment on acute bone infection were both investigated.

## Materials and methods

### Bacterial strain and selection of antibiotic

The bacterial strain selected in the study was the ATCC-MSSA 29213 standard strain, which is one of the most common causative pathogens of bone infection and is resistant against many classes of antimicrobials [13]. Given that the purpose was to investigate the effect of systemic antibiotic treatment together with MWs, we chose cefuroxime, which can reach a certain concentration in bone tissue [14]. The minimum inhibitory concentration (MIC) of cefuroxime for the tested strain is 0.5–2 mg/ml. After 24 h of incubation of the isolate at 37 °C (Thermo-3111, Thermo Scientific, USA), several colonies were added into sterile saline to form a 1 × 10^8^ colony-forming units (CFU)/ml MSSA suspension, corresponding to a turbidity of 0.5 McFarland standards (DensiCHEK Plus, BioMérieux, Inc., USA).

### Animals and surgical procedure of acute bone infection

Forty male rats at approximately the same age (280–310 g) were chosen for this study. All animal experiments were conducted in accordance with the approval of the Institutional Committee on the Care and Use of Animals of Nanjing Drum Tower Hospital, Nanjing University Medical School (issue 2018/04). For acclimatization, animals were fed standard rat chow and water and housed in cages with controlled temperature and a 12-h light/dark cycle for at least 1 week before the experiment.

The experimental model performed in this study was primarily based on the procedure described by Fukushima et al. [15]. Acute bone infection was induced in the tibiae under general anesthesia by weight-adapted intraperitoneal injection of 10% chloral hydrate, 350 mg/kg body weight (BW). Ibuprofen was given preemptively at the onset of surgery. Animals were prepared for surgery as follows: The right leg was shaved, and the site of operation was surgically sterilized and draped. Strict asepsis was required during the surgical procedure to avoid other microbial contamination. A 1.5-cm longitudinal incision was made at the proximal metaphysis in the anterior tibia, and a hole was drilled through the cortex using a high-speed drill with a 0.7-mm-diameter bit. The medullary cavity contents were extracted using a 1-ml injection syringe, and then, 0.1 ml of the 1 × 10^8^ CFU/ml MSSA suspension was injected into the hole by an insulin injector. Afterward, the hole was sealed with bone wax. Finally, the fascia and skin were closed with sutures in sequence and disinfected.

Postoperatively, the animals received ibuprofen (15 mg/kg BW daily) to control postoperative pain for 2 days. The rats were closely followed, and the wounds were observed every day. Their behaviors as well as weight were monitored regularly.

### Treatment modalities

All animals were randomized and assigned to four groups as follows: group I was acute bone infection + saline (control); group II, acute bone infection + saline + MW therapy; group III, acute bone infection + systemic cefuroxime; and group IV, acute bone infection + systemic cefuroxime + MW therapy.

Saline and cefuroxime were administered immediately after the induction of acute bone infection, and MW therapy was conducted approximately 2 h after injection of saline and cefuroxime. Groups I and II received saline (30 ml/kg BW) daily. In groups III and IV, the daily dose of cefuroxime was 30 mg/kg BW. Saline and cefuroxime were administered by the intraperitoneal route.

**Fig. 1:**
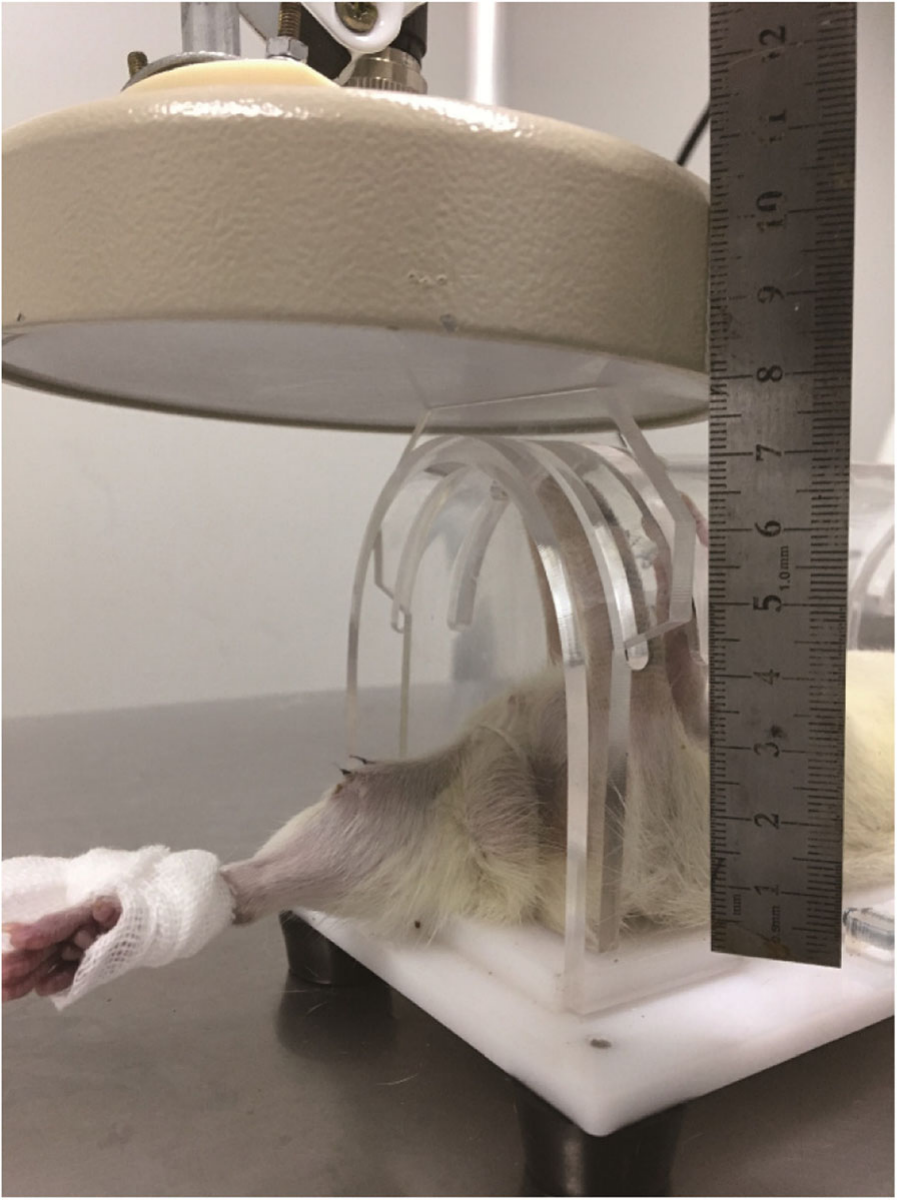
The right leg of the rat underwent MW exposure

The animals in groups II and IV received MW treatment every day after surgery. MW treatment was performed under general anesthesia. Only the right tibia received MW treatment (Fig. 1). The applicator was connected to a 2450 MHz-MW generator (WB-3100AI, Baoxin Medical Equipment Co., Ltd., Xuzhou, China) with power output ranging from 0–100 W. A 25-W continuous-wave MW exposure was used. The MW treatment lasted for 20 min per day. The non-contact applicator was parallel to the lesion and 8 cm away from the skin (Fig. 1).

**Fig. 2:**
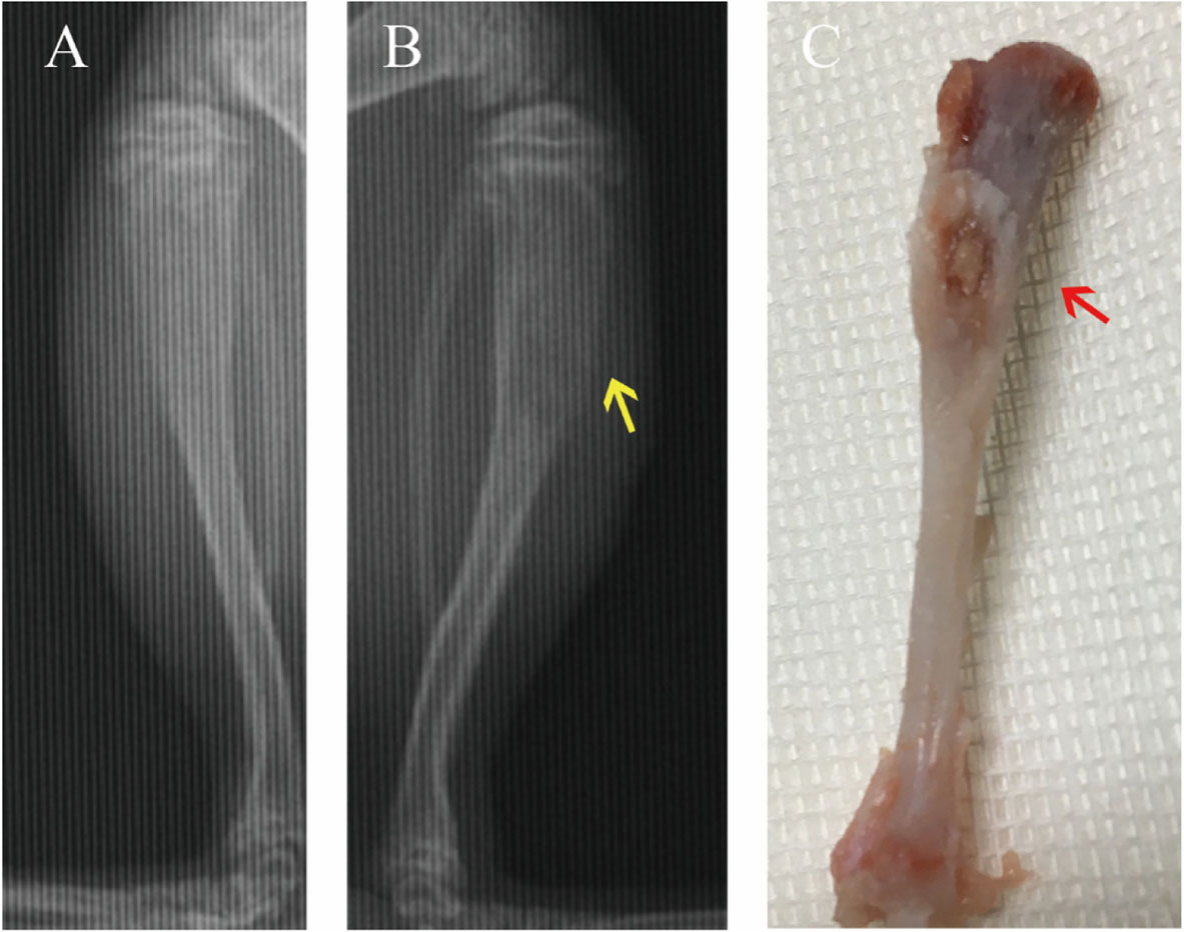
Signs of bone infection in the control group. Skeletal changes were obscure on X-rays, but osteolytic change and bone destruction (yellow arrow) still could be seen (**b**) compare to the contralateral tibia (**a**); biopsy specimen showed pus (red arrow) on the proximal tibia (**c**)

After 7 days, radiographs were taken with rats in the prone position, and all rats were weighed and then sacrificed via rapid cervical dislocation. The tibiae were dissected and retrieved under sterile conditions for microbiologic analysis. The establishment of bone infection could be seen in the control group (Fig. 2).

### Microbiologic analysis

Biopsy specimens of the infected tibiae were analyzed by microbiology procedures, and the microbiologist was blinded to the groups. Standard plate count analysis was performed. The tibiae were fragmented, and 10 ml of sterile saline was added to a 15-ml sterile centrifuge tube. The centrifuge tube was vortexed and homogenized, and then, 10-fold serial dilutions of 10^–1^ to 10^–3^ of the samples were prepared using sterile saline to accurately count the number of bacteria. Then, 200 μl of each of these suspensions was streaked onto Columbia Blood Agar Base Medium (BioMérieux, Inc., USA) in duplicate. After 24 h of incubation of the blood agar plates at 37 °C (Thermo-3111, Thermo Scientific, USA), the colonies on the plates were counted and calculated as per milliliter of suspension (CFU/ml). Final identification was performed with a Vitek™ 2 compact system (BioMérieux, Inc., USA) for confirmation.

### Statistical analysis

As the data were skewed, log transformation was applied for all the variables. All calculations were carried out using SPSS 22.0 (SPSS, Inc., Chicago, IL). Quantitative culture data are expressed as the mean and standard deviation (SD) of CFU. For group comparison, the analysis of variance (ANOVA) test was used. A *p* value less than 0.05 was considered significant.

## Result

### Quantitative culture of excised tissues

No abnormal behavioral patterns, such as fatigue and aggressiveness, were observed among the rats during the course of these experiments. No animal displayed severe skin infection or died before sacrifice. The rats in groups I, II, and IV lost weight (mean 21 g, 31.7 g, and 13.6 g, respectively), while the animals in the antibiotic group gained weight (mean 4.8 g). The *p* values of weight changes between the groups are shown in Table 1.

**Fig. 3:**
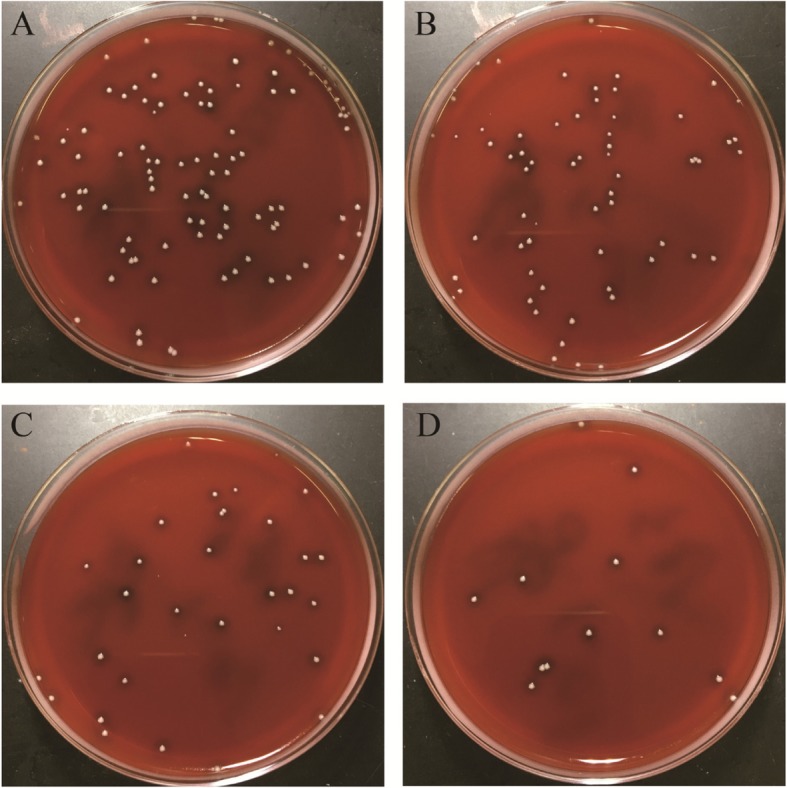
Typical bacterial colonies grown on the blood agar plates (at serial dilutions of 10–3). **a** Group I without treatment. **b** Group II receiving only MW therapy. **c** Group III receiving only systemic cefuroxime. **d** Group IV treated with a combination of MW and systemic cefuroxime

Table 2 presents the bacterial counts; compared with that in group I, the mean percent decrease in bacterial counts was 17.9% in group II, 56.4% in group III, and 89.7% in group IV. A significantly reduced bacterial count was found in group IV compared with those in the other three groups (Fig. 3). A statistically significant difference was noted between groups III and IV. Additionally, the mean bacterial number of group II was lower than that of group I, but no significant difference was detected. Table 3 lists the *p* values of bacterial counts in paired comparisons of treatment groups.

**Table 1:** *p* values between the groups according to weight changes

Group	Control	MW	AB
MW	0.223	–	< 0.001^a^
AB	0.005^a^	< 0.001^a^	–
MW+AB	0.392	0.043^a^	0.04^a^

*MW* microwave, *AB* antibiotic

^a^The mean difference is significant at the 0.05 level

**Table 2:** Mean bacterial loads in treatment groups

Groups	*n*	CFU/ml(× 10^5^)		Log CFU/ml	
		Mean	SD	Mean	SD
Control	10	4	1.5	5.6	0.2
MW	10	3.2	1.7	5.5	0.2
AB	10	1.7	1.1	5.1	0.5
MW + AB	10	0.4	0.5	4.4	0.5

*MW* microwave, *AB* antibiotic, *SD* standard deviation

## Discussion

To our knowledge, this study is the first to demonstrate that MWs play a role in the treatment of bone infection in vivo. In this study, an acute osteomyelitis model was produced using an MSSA strain. One week after initiation of treatment, bacterial counts in group IV (cefuroxime + MW) decreased significantly compared to those in the untreated control group, group II (MW) and group III (cefuroxime). These results demonstrate that cefuroxime + MW significantly reduced bacterial counts and that MW therapy provides significant synergy for systemic antibiotic treatment. In our study, the average bacterial counts in the “only MW” group were lower than those in the control group, although the difference was not statistically significant. Therefore, we concluded that the MW therapy did not promote bacterial proliferation or increase the spread of bacteria.

MW energy has been reported to be bactericidal to gram-positive and gram-negative bacteria in vitro [12, 16]. In our study, the combination of cefuroxime + MW significantly reduced bacterial counts; two possible explanations account for this phenomenon. First, the temperature of saline in vitro can be achieved and maintained at 45 °C under 2450-MHz-MW radiation for 20 min, and as the tissue temperature increases from 41 to 45 °C, the blood perfusion increases by approximately 15 times [11]. This change can increase the local microcirculation in the irradiation area, which can heighten both the local antibiotic concentration and the anti-inflammatory or immune response [17] to achieve local or systemic bactericidal or bacteriostatic effects. In addition, hyperthermia could produce an increase in nutrients and oxygen in the heated region, which contributes to the effect of tissue repair. In addition, MWs also influence cytoarchitecture. Shamis et al. [18] used two microscopic analyses to evaluate the effects of MW radiation on *E. coli* cells at sub-lethal bulk temperatures, and the results suggested that temporary pores were formed within the cell membrane, resulting in a substantial increase in cellular permeability to ions and molecules during MW exposure. The pores in the cell membrane may make the bacteria more sensitive to antibiotics.

The results of weight changes demonstrated that there was a significant difference in the decrease in rat weight in group II (MW) compared to that in group III (cefuroxime) and group IV (cefuroxime + MW). Furthermore, a significant difference was found between group III (cefuroxime) and group IV (cefuroxime + MW), indicating that MWs may have an effect on rats to some extent. Hyperthermia caused by MWs induces a general increase in the metabolic rate [19]. A previous study reported that the biological effects caused by MWs were correlated with the power density, frequency, waveform, modulation, and duration of exposure [20]. Another study found that prolonged whole-body MW exposure of rats to a 2.45-GHz frequency (2 h per day for 35 days) adversely affects the organs, including the brain, liver, kidney, spleen, and testicular organs [21, 22]. In the present study, 2450-MHz-frequency continuous-MW exposure (20 min per day for 7 days) was used, and the temperature of saline in vitro was maintained at 45 °C under these conditions. This method corresponds to the practice that the temperature should not exceed 45 °C for 30 min in musculoskeletal medicine [11]. No abnormal behavioral patterns or wound-related incidents were observed postoperatively. Therefore, we infer that MW therapy is relatively safe to apply under the condition of a 2450-MHz frequency for 20 min per day (8 cm away from the skin). A histologic assessment should be conducted for further evaluation.

There are several limitations in the current study. First, staphylococci were selected in this study because they are the predominant bacteria in bacterial contamination of open fractures [23], but additional bacterial strains need to be investigated. Second, the small sample size of rats is another limitation of this study. Last but not the least, considering that the application of this treatment prevented bone infection in this study, further studies are needed to confirm its effect in curing clinical cases.

**Table 3:** *p* values of bacterial counts in paired comparisons of treatment groups (log CFU/ml)

Group	Control	MW	AB
MW	0.545	–	0.034^a^
AB	0.008^a^	0.034^a^	–
MW+AB	< 0.001^a^	< 0.001^a^	< 0.001^a^

*MW* microwave, *AB* antibiotic, *SD* standard deviation

^a^The mean difference is significant at the 0.05 level

## Conclusion

Based on the results above, the present study suggests that MW therapy may be a valuable auxiliary tool in the management of acute osteomyelitis, that it acts synergistically with systemic antibiotic treatment, and that this combination is safe in rats.

## Data Availability

All data generated or analyzed during this study are provided within this article.

## Abbreviations

AB: Antibiotic
ANOVA: Analysis of variance
BW: Bodyweight
CFU: Colony forming units
MHz: Megahertz
MIC: Minimum inhibitory concentration
MSSA: Methicillin-sensitive *Staphylococcus aureus*
MW: Microwave
SD: Standard deviation
